# Remission in Type 2 Inflammatory Diseases: Current Evidence, Unmet Needs, and Suggestions for Defining Remission in Chronic Rhinosinusitis with Nasal Polyps

**DOI:** 10.1007/s11882-023-01118-6

**Published:** 2023-12-12

**Authors:** Marco Caminati, Eugenio De Corso, Giancarlo Ottaviano, Carlotta Pipolo, Michele Schiappoli, Veronica Seccia, Francesca Romana Spinelli, Edoardo Vincenzo Savarino, Paolo Gisondi, Gianenrico Senna

**Affiliations:** 1 Department of Medicine, University of Verona, Verona, Italy; 2 Allergy Unit and Asthma Center, Verona University Hospital, Verona, Italy; 3 Otorhinolaryngology, Head and Neck Surgery, “A. Gemelli” Hospital Foundation IRCCS, do A. Gemelli 8, 00168 Rome, Italy; 4 Department of Neurosciences, Otolaryngology Section, University of Padova, Padua, Italy; 5 Otorhinolaryngology Unit, ASST Santi Paolo e Carlo, Department of Health Sciences, Università Degli Studi di Milano, 20142 Milan, Italy; 6 Otolaryngology Audiology and Phoniatric Operative Unit, Department of Surgical, Medical, Molecular Pathology and Critical Care Medicine, Azienda Ospedaliero Universitaria Pisana, University of Pisa, 56124 Pisa, Italy; 7 Rheumatology Unit, Department of Clinical Internal, Anesthesiology and Cardiovascular Science – Sapienza University of Rome, Piazzale Aldo Moro 5, 00185 Rome, Italy; 8 Department of Surgery, Oncology and Gastroenterology, University of Padua, Padua, Italy; 9 Gastroenterology Unit, Azienda Ospedale Università di Padova, Padua, Italy; 10 Dermatology and Venereology, Department of Medicine, University of Verona, Verona, Italy

**Keywords:** Asthma, Atopic dermatitis, Biological therapy, Chronic rhinosinusitis with nasal polyps, Eosinophilic esophagitis, Remission, Type 2 inflammatory disease

## Abstract

**Purpose of Review:**

The development of biological therapies for type 2 inflammatory diseases raises the possibility of addressing remission in those dis-immune conditions. No consensus exists for a definition of remission in chronic rhinosinusitis with nasal polyps (CRSwNP). This review aims to critically evaluate the published data to provide the basis for defining remission in CRSwNP.

**Recent Findings:**

The published evidence has yet to provide an unequivocal definition on remission in type 2 inflammatory diseases, in part reflecting differences in approaches to diagnosis and follow-up. A multidimensional evaluation is necessary when considering complete remission, including clinical, inflammatory, and histologic criteria, but how to combine or tailor the three perspectives according to disease severity at baseline or timing of assessment of treatment category is yet to reach consensus. We suggest defining remission starting from the approach taken in asthma and eosinophilic esophagitis, that is, including the resolution of symptoms and improvements in objective parameters of disease severity and/or inflammatory activity. Future studies and consensuses should provide validated criteria with cutoffs for the day-to-day definition of remission.

**Summary:**

The definition of remission in CRSwNP should include the following criteria, to be verified and maintained for a period of ≥ 12 months: absence of symptoms (nasal obstruction, loss of smell, rhinorrhea as the main ones); no impact of symptoms on quality of life; no need of surgery; no chronic or rescue medications (systemic corticosteroids or antibiotics); and recovery of smell function, possibly evaluated by objective test. Assessment of underlying inflammation should also be considered once accurate and feasible biomarkers are available in clinical practice.

## Introduction

Prior to the development of targeted biological therapy for allergic diseases, the goals of treatment were the reduction of symptoms and flares/complications and the restoration or maintenance of function in the affected organ [1–5]. Biological therapy has revolutionized the treatment of many chronic inflammatory diseases, including psoriasis, rheumatoid arthritis, and inflammatory bowel disease, allowing for the development of a “treat-to-target” approach that ultimately aims at achieving and maintaining the target (remission) in many patients [6–8]. In rheumatic diseases, the first definition of remission dates back to 1996 [9]; later on, with the spread of the treat-to-target strategy, it became evident that the achievement of the target was associated with an improvement in short- and long-term clinical outcomes and patients’ quality of life, with not negligible economic benefits due to less direct and indirect costs for the management of the disease [10].

The development of biological therapies for type 2 inflammatory diseases now raises the possibility of addressing remission in those dis-immune conditions. For remission to be included as a goal of treatment, the term “remission” must be clearly defined; however, this has not been the case in most type 2 inflammatory conditions. In fact, an agreement on asthma remission has not yet been reached, and a definition is lacking in the case of chronic rhinosinusitis with nasal polyps (CRSwNP).

This narrative review aims to critically evaluate the currently available literature regarding the definition of remission across type 2 inflammatory diseases, seeking new insight as the basis for defining remission in patients with CRSwNP.

## Methods

A literature search of the PubMed database was undertaken to identify English language papers published in indexed journals up to August 2023 according to the following research keywords: severe asthma AND remission, atopic dermatitis AND remission, eosinophilic esophagitis (EoE) AND remission, and CRSwNP AND remission. We excluded case reports, correspondence, editorials, and non-English language articles. Only articles including a detailed definition of remission or at least a focus on the issue were considered. The search results were supplemented with additional literature identified ad hoc or via the bibliographies of identified studies.

## Existing Definitions of Remission in Type 2 Inflammatory Diseases

### Atopic Dermatitis

One systematic review was identified that defined remission in patients with atopic dermatitis (AD) [11]. This review, which investigated predictive factors for complete remission of infant-onset AD, defined remission in treated patients as the absence of signs/symptoms of AD as assessed by a physician (clinical assessment) or by the International Study of Asthma and Allergies in Childhood (ISAAC) questionnaire in at least two subsequent follow-up visits covering a period of ≥ 12 months. The main outcome of interest was achievement of complete on-treatment remission of infant-onset AD before the age of 6–7 years; remission of AD in older children (i.e., aged 10, 11–14, or 15–17 years) and adults (> 18 years) was also evaluated [11]. These authors noted that there is no consensus regarding the definition of complete remission in AD, with remission being reported as either “no AD after the age of 2 years” or “not having a specific allergy-related disease that had been presented at the previous follow-up visit” [11].

Another systematic review has investigated different strategies for achieving long-term disease control in adults or children with AD, but this study did not provide a definition of remission and noted a lack of consensus on how to measure long-term disease control in AD [12]. Some of the measures used to determine long-term disease control were analysis of AD flares, use of AD medications, the Scoring Atopic Dermatitis (SCORAD) scale, quality of life scales, pruritus scales, percentage of affected body surface area, Eczema Area and Severity Index (including modified version), and Investigator Global Assessment [12].

### Eosinophilic Esophagitis

Eight studies have provided definitions for on-treatment remission in EoE (Table 1). These included six randomized controlled trials (RCTs) [13–16, 17•, 18], one open-label extension of an RCT [19], and one prospective cohort study [20]. Two studies were conducted in children [14, 20], two were in children and adults (aged ≥ 12 years in one [18] and aged 3–30 years in the other [13], and four were in adults [15, 16, 17•, 19]. In addition, two systematic reviews have assessed the definitions for remission in EoE across various studies [21, 22]. Three studies provided definitions for histological remission, two for clinical or symptomatic remission, and one for complete remission, encompassing both histological and symptomatic components.

**Table 1:** Definitions of remission in eosinophilic esophagitis from articles published in the last 10 years

**Article**	**Article type**	**Definition of remission**
**Children**		
Gupta et al. [14]	RCT	Histologic remission: PEC ≤ 1/HPF in all oesophageal levels (proximal, mid, and distal) Symptom resolution: EoE Clinical Symptom Score 0
Collins et al. [20]	Prospective cohort study	Histologic remission: EoEHSS score ≤ 3 (both grade and stage scores) *plus* scores defining PEC < 15/HPF
**Adults or children**		
Butz et al. [13]	RCT	Complete remission: eosinophils ≤ 1/HPF
Dellon et al. [18]	RCT	Histologic remission: intraepithelial PEC ≤ 6/HPF
**Adults**		
Miehlke et al. [15]	RCT	Histologic remission: eosinophils mean < 16/mm^2^ of HPF
Lucendo et al. [16]	RCT	Complete remission: NRS dysphagia and odynophagia score ≤ 2 (on a 0–10 scale) on each of the 7 days before the end of the DB phase *plus* a PEC < 5/HPF
Strauman et al. [17•]	RCT	Clinical remission: NRS dysphagia and odynophagia score ≤ 2 (on a 0–10 scale) on each of the last 7 days of induction therapy Histologic remission: PEC < 16/mm^2^
Dellon et al. [19]	Open-label extension of RCT	Symptomatic remission: EEsAI score ≤ 20

*DB* double-blind, *EEsAI* Eosinophilic Esophagitis Activity Index, *EoEHSS* Eosinophilic Esophagitis Histology Scoring System, *HPF* high-powered field, *NRS* Numerical Rating Scale, *PEC* peak eosinophil count, *RCT* randomized controlled trial

Histological remission was variously described as an intraepithelial peak eosinophil count (PEC) of < 16 per high-powered field (HPF) [18], a PEC of < 16 per mm^2^ of HPF (considered to be equivalent to < 5 eosinophils per HPF) [17•], or the combination of an Eosinophilic Esophagitis Histology Scoring System (EoEHSS) score of ≤ 3 for both grade and stage plus scores defining a PEC < 15 per HPF [20]. The systematic review by Eke and colleagues also noted that the definition of histological remission differed between clinical studies but that 80% of reviewed RCTs defined remission in terms of a PEC of between 0 and ≤ 5 per HPF [21]. This was also the definition used in 80% of reviewed studies investigating the effectiveness of monoclonal antibody (mAb) treatment in patients with EoE. In studies of proton pump inhibitors or diet elimination treatment formulas, the definition of remission was more variable. In these studies, remission was defined as 0 to ≤ 5 eosinophils per HPF, 11 to ≤ 15 per HPF, or ≤ 15 per HPF [21, 22]. It was also noted that definitions based only on the PEC might miss histological features that are indicative of unresolved EoE (even in the presence of low eosinophil counts), such as eosinophilic micro-abscesses, basal cell hyperplasia, or extracellular eosinophil granules [21]. In this respect, a definition of histological remission that encompasses the EoEHSS score may be more appropriate than one based purely on the PEC. Evidence of remission in more than one level of the esophagus may also be required, as was used in the definitions by Butz and colleagues [13] and Gupta and colleagues [14].

In the literature we identified, clinical or symptomatic remission was defined as an Eosinophilic Esophagitis Activity Index (EEsAI) score of ≤ 20 [19] or dysphagia and odynophagia severity score of ≤ 2 (on a numerical rating scale [NRS] of 0–10) on each day of the last week of induction therapy [17•] or a reduction of > 90% in the Dysphagia Symptom Questionnaire (DSQ) score [23, 24]. Complete remission was defined as the combination of low symptoms and low eosinophil infiltration in biopsy samples, specifically dysphagia and odynophagia severity score of ≤ 2 (on an NRS of 0–10) on each of the 7 days before the end of the double-blind phase plus a PEC of < 5 per HPF [16].

In patients with EoE, there is a low correlation between symptoms and endoscopic or histological features [25]; therefore, a combination of both features is required to obtain a clinically meaningful definition of disease activity [26]. In terms of the measures used, the EoEHSS has been recommended for histological assessment based on its validity, while the EEsAI is recommended for symptomatic assessment in adults based on its validity and responsiveness [27, 28].

### Asthma

Of the 14 studies identified that focused on the definition of asthma remission (Table 2), 10 were longitudinal cohort studies, [29–38] one was a post hoc analysis of phase 3 studies [39], and two were a consensus report [40, 41]. In addition, a consensus for asthma remission using a Delphi method and involving a panel of experts within the Severe Asthma Network Italy (SANI) has been recently published [42•].

**Table 2:** Definitions of remission in asthma from articles published in the last 10 years

**Article**	**Article type**	**Definition of remission**
**Children**		
Javed et al. [29]	Retrospective cohort study	Absence of signs/symptoms of asthma for ≥ 3 consecutive years without (1) asthma signs/symptoms according to the clinician’s medical record; (2) patient use of asthma medications; (3) clinic, urgent care or ED visits for asthma symptoms; and (4) hospitalization for asthma
**Adults**		
Cazzoletti et al. [30]	Prospective cohort study	No current use of asthma medications, no asthma-like symptoms (wheezing, tightness in the chest or shortness of breath) and no asthma attacks in the past 12 months
Wu et al. [31]	Prospective cohort study	Absence of symptoms for > 3 years without relapse and without asthma medicine in the past 1 year
Sӧzener et al. [32]	Retrospective and prospective cohort study	Clinical remission: no asthma symptoms and no use of asthma treatment for ≥ 2 years Complete remission: clinical remission plus normal bronchial provocation tests
Tuomisto et al. [33]	Prospective cohort study	Absence of symptoms and ACT score < 25 without any asthma medication for the past 6 months and no oral prednisolone in the past 2 years; additional criteria were normal lung function (prebronchodilator FEV_1_ > 80% and FEV_1_/FVC ratio > 0.7), bronchodilator response < 12%, FEV_1_ 200 mL and FeNO ≤ 20 ppb
Westerhof et al. [34]	Prospective cohort study	Absence of symptoms for ≥ 1 year without relapse and without asthma medicine in the past ≥ 1 year
Almqvist et al. [35]	Prospective cohort study	Absence of any wheeze or attacks of shortness of breath and no asthma medications in the past 1 year
Qi et al. [36]	Prospective cohort study	Clinical remission: (1) without any asthma medication; (2) no symptoms (asthma attacks and/or wheezing) in the past 1 year Complete remission: clinical remission criteria (1) and (2) above; plus (3) no AHR; and (4) prebronchodilator FEV_1_ > 80% predicted
Menzies-Gow et al. [40]	Consensus report	Clinical remission: cessation of significant symptoms for a specified period of time^a^ and absence of systemic corticosteroid-requiring attacks for a specified period of time^a^ Complete remission: cessation of significant symptoms and inflammation; absence of AHR (in research settings only)
Tupper et al. [37]	Longitudinal cohort study	Clinical remission: (1) no asthma symptoms (wheeze, dyspnoea, chest tightness, cough and/or sputum) in the past 1 year; (2) no currently prescribed and self-reported use of asthma medications in the past 1 year Complete remission: clinical remission plus (1) FeNO < 50 ppb; (2) no bronchodilatory reversibility; (3) no AHR; or (4) spirometry with FEV_1_ ≥ 80% predicted and FEV_1_/FVC ratio ≥ 0.70 (if not the only factor)
Menzies-Gow et al. [39]	Post hoc analysis of phase 3 studies	Composite of (1) no exacerbations; (2) no OCS use; (3) ACQ-6 score < 1.0 or ≤ 0.75; and (4) prebronchodilator FEV_1_ increase from baseline of ≥ 100 mL at 6 or 12 months
Numata et al. [38]	Retrospective study	Composite of (1) no exacerbations requiring OCS for 12 months; (2) no maintenance OCS; (3) ACT score ≥ 20; and (4) FEV_1_ ≥ 80% predicted
Lommatzsch et al. [41]	Consensus report	Sustained absence of asthma symptoms and exacerbations Sustained absence of asthma exacerbations Stable lung function No need for SCS for the treatment of asthma
Canonica et al. [42•]	Delphi consensus report	No further need for oral corticosteroids and all three of the following criteria are met for ≥ 12 months: absence of asthma symptoms; absence of asthma exacerbations/attacks; stable lung function

*ACQ* Asthma Control Questionnaire, *ACT* Asthma Control Test, *AHR* airway hyper-responsiveness, *ED* emergency department, *FeNO* fraction of exhaled nitric oxide, *FEV*_*1*_ forced expiratory volume in 1 s, *FVC* forced vital capacity, *OCS* oral corticosteroids, *ppb* parts per billion, *SCS* systematic corticosteroids

^a^Exact period of time not yet defined [40]

As for EoE, the definitions for remission of asthma in adults and children encompassed both clinical and inflammatory components. In general, the clinical definition of remission included the absence of symptoms with or without asthma attacks and without the use of asthma medication [29–37, 40], but the specific criteria varied considerably between studies. Within the definition of remission, the required duration of no symptoms plus no medication ranged from 6 months [39] to ≥ 3 years [29, 31] but was most commonly 12 months [30, 34–38]. Most studies required that patients had no symptoms and/or asthma attacks without the use of asthma medication [29–37, 40], implying that this is a “true” remission in those patients who no longer need treatment. However, two studies defined remission as no or low symptoms/exacerbations and no use of oral corticosteroids (OCS) rather than no use of any asthma medication [38, 39]. The latter two definitions are consistent with the recommended definition of asthma remission in the consensus report by Menzies-Gow and colleagues [40]. This report suggested that one criterion for clinical remission should be “*the absence of significant symptoms for a specified period of time* (*exact duration to be defined*)” and that another criterion should be “*the absence of systemic corticosteroid-requiring attacks for a specified period of time* (*exact duration to be defined*)” [40]. This consensus did not require the definition to be the absence of symptoms/exacerbations *plus* the absence of asthma medication, implying that remission can be achieved while still receiving treatment. This definition appears to be the best choice for studies investigating the effectiveness of asthma medication since it does not require patients to be symptom-free without treatment.

Three studies [32, 36, 37] and the consensus report [40] included two definitions of remission: clinical (i.e., the absence of symptoms/flares) and complete (i.e., clinical remission plus the absence of lung function impairment). Three further studies included a lung function component in the definition of remission but did not specify this as “complete remission” [33, 38, 39].

Where lung function criteria were included in the definition of remission, these criteria were a forced expiratory volume in 1 s (FEV_1_) of ≥ 80% predicted [33, 37, 38], an increase in prebronchodilator FEV_1_ by ≥ 100 I compared with baseline [39], an FEV_1_/forced vital capacity (FVC) ratio of ≥ 0.7 [33, 37], a fraction of expired nitric oxide (FeNO) of ≤ 20 parts per billion (ppb) [33] or < 50 ppb [37], no bronchodilator reversibility [33, 37], and no airway hyperresponsiveness (AHR) [32, 36, 37]. Other authors have suggested that a blood eosinophil count of < 300 cells/μL, a sputum eosinophil count of < 3%, and a reduction in subepithelial fibrosis are potential proofs of normalized airway pathology [43]. The consensus report by Menzies-Gow and colleagues recommends including the absence of AHR as a criterion for complete remission in a research setting but notes that this may not be feasible in routine clinical practice [40]. In a review by Rial and Domínguez-Ortega, it was noted that some degree of AHR or lung function impairment may be present in patients with asthma without significant symptoms or in those with low or undetectable serum or airway biomarker levels (e.g., FeNO, eosinophils, or allergen-specific immunoglobulin [Ig]E) [44]. According to these authors, remission in asthma can be defined in three ways: (1) clinical remission, defined as no significant symptoms or the use of corticosteroid medications for ≥ 1 2 months with improved lung function tests; (2) inflammatory remission, defined as very low or undetectable airway or serum biomarker levels (such as eosinophils, allergen-specific IgE, periostin, FeNO); or (3) complete remission, defined as the absence of asthma symptoms without the use of medication. Only patients with complete remission would no longer show signs of bronchial hyper-responsiveness [44].

A recent independent expert opinion-based definition identified four main criteria for clinical disease remission in asthma: sustained absence of asthma symptoms, sustained absence of asthma exacerbations, stable lung function, and no need for systemic corticosteroids (SCS) for the treatment of asthma for at least 12 months. The authors also remarked that relying on the evidence available so far, only on treatment clinical remission is achievable both with traditional inhaled therapies and with biologic drugs, their discontinuation resulting in disease worsening. Thus, asthma remission should be considered a pragmatic and achievable therapeutic aim [41].

The recent definition of on-treatment asthma remission by SANI relied on a Delphi method study including the experts from the referral centers belonging to Severe Asthma Network Italy [42•]. Two sets of criteria were established: complete clinical remission criteria, including the absence of need for OCS, the absence of symptoms, the absence of exacerbations/attacks, and pulmonary stability, and partial clinical remission criteria, including the absence of need for OCS, and 2 out of the 3 following criteria: the absence of symptoms, the absence of exacerbations/attacks, and pulmonary stability.

In addition, the duration of the abovementioned conditions has to be verified for at least 1 year to fulfill the remission definition. No complete consensus was reached about specific tools and/or cutoffs to assess remission in terms of clinical, functional, inflammatory, and quality of life-related parameters.

### Definition of Remission in CRSwNP

Remission in CRSwNP has not been defined in the literature so far. However, with the advent of new biologic drugs, a resolution of symptoms and improvement of endoscopic findings of the disease have been observed over time, suggesting that biologics may lead to a clinical remission of the disease under treatment.

Randomized clinical trials of biological therapies in patients with CRSwNP, although not explicitly focusing on remission, provide some insights about concepts and tools that might be useful in defining it. The RCTs (i.e., benralizumab in OSTRO [45•], dupilumab in SINUS-24 and SINUS-52 [46•], and mepolizumab in SYNAPSE [47•]) have used several endpoints to define clinical outcomes (Table 3). All of these studies adopted as the primary endpoint change from baseline in nasal polyp score (NPS) in combination with nasal congestion severity (NCS) or visual analog scale (VAS) nasal obstruction [45•, 46•, 47•]. Similarly, with the exception of Kilty and Lasso 2022 [48], which had the 22-Item Sino-Nasal Outcome Test (SNOT-22) as its primary endpoint, real-world studies have had NPS or a combination of NPS and change in nasal obstruction as primary endpoints [49•, 50••, 51•, 52•, 53•, 54, 55•]. Secondary endpoints included improvements in symptoms, quality of life (measured by the SNOT-22), Lund-Mackay score (computed tomography [CT]), peak nasal inspiratory flow, and the need for surgery or systemic therapy.

**Table 3:** Endpoints used in studies of biological therapies in patients with chronic rhinosinusitis with nasal polyps

**Randomized controlled studies**	**Co-primary endpoint**	**Secondary endpoints**						
		**Symptoms**^**a**^	**Smell**	**HR-QoL**	**CT imaging**	**Other treatment**	**Nasal patency**	**Other**
OSTRO (benralizumab) [45•]	Change in NPS + NBS	Scale of 0 to 3	DSS	SNOT-22	Lund-Mackay score	Nasal surgery or OCS use	–	–
SINUS-24 and SINUS-52 (dupilumab) [46•]	Change in NPS + NCS	Scale of 0 to 3	UPSIT	SNOT-22	Lund-Mackay score	Nasal surgery or OCS use	Nasal PIF	Rhino-sinusitis VAS FEV_1_ ACQ-6
SYNAPSE (mepolizumab) [47•]	Change in NPS + nasal obstruction VAS	VAS	VAS	SNOT-22	–	–	Nasal PIF	–
POLYP 1 AND POLYP 2 (omalizumab) [66]	Change in NPS + NCS	VAS	UPSIT	SNOT-22	–	Nasal surgery or OCS use	–	–
**Real-world studies**								
Meier et al. (omalizumab, mepolizumab, benralizumab, dupilumab) [49•]	Change in NPS + nasal obstruction	Change in subjective symptoms	NS	–	–	Nasal surgery or OCS use	–	–
De Corso et al. (dupilumab) [50••]	Change in NPS + nasal obstruction	VAS	Sniffin’ Sticks VAS olfaction	SNOT-22 and EQ-VAS		Nasal surgery or OCS use	Nasal PIF	–
Haxel et al. (dupilumab, omalizumab) [51•]	NPS	VAS	Sniffin’ Sticks	SNOT-22	–	Nasal surgery or OCS use	–	–
Kilty and Lasso (dupliumab) [48]	SNOT-22	–	–	SNOT-22	–	–	–	–
Nettis et al. (dupliumab) [52•]	Change in NPS + nasal obstruction	VAS	VAS	SNOT-22 RQLQ	–	Nasal surgery or OCS use	–	Spirometry, ACT, and other asthma and allergy tests
Ottaviano et al. (dupliumab) [53•]	Change in NPS + nasal obstruction	VAS	Sniffin’ Sticks VAS olfaction	SNOT-22	–	Nasal surgery or OCS use	Nasal PIF	ACT
Trimarchi et al. (dupliumab) [54]	Change in NPS	VAS	B-SIT	SNOT-22	–	Nasal surgery or OCS use	–	ACT
Jansen et al. [55•]	Change in NPS + nasal obstruction	VAS	Sniffin’ Sticks	SNOT-22	–	Nasal surgery or OCS use	–	Rhino-sinusitis VAS FEV_1_

*ACQ-6* 6-Item Asthma Control Questionnaire, *ACT* Asthma Control Test, *B-SIT* Brief UPSIT, *CT* computed tomography, *DSS* difficulty with sense of smell score from 0 (absent) to 3 (severe), *FEV*_*1*_ forced expiratory flow in 1 s, *NBS* Nasal Blockage Score from 0 (absent) to 3 (severe), *NCS* Nasal Congestion Severity, *NPS* Nasal Polyp Score, *NS* not stated, *OCS* oral corticosteroids, *PIF* peak inspiratory flow, *RQLQ* Rhinoconjunctivitis Quality of Life Questionnaire, *SNOT-22* 22-Item Sino-Nasal Outcome Test; *UPSIT* University of Pennsylvania Smell Identification Test, *VAS* visual analog scale from 0 to 10 [46•] or 0 to 10 cm [47•]

^a^Blockage/obstruction, nasal discharge, postnasal drip, loss of smell, and composite total

The primary endpoints were predominantly in line with recommendations of the US Food and Drug Administration (FDA) that proposed to encompass both endoscopic assessment of nasal polyps (with the NPS as the preferred instrument) and assessment of a patient-reported nasal symptom score [56]. On the other hand, despite the use of the SNOT-22 score being common in RCTs, the FDA recommends against the use of SNOT-22 (or other versions of SNOT) as the primary study endpoint in CRSwNP registration trials because of inherent concerns about its interpretability and the redundancy of some SNOT-22 items with other symptom scales [56].

For all of these reasons, these outcome tools have entered into routine clinical practice as parameters for evaluating the success of biological therapy. In 2021, EUFOREA set out to define “adequate response” in a multi-parametric way using some cutoffs as follows: NPS of < 4, a nasal congestion score of < 2, a total symptom VAS score of < 5, a SNOT-22 score of < 30, and no current need for nasal surgery or SCS after 12 months of therapy [57•]. Nevertheless, De Corso et al., in the phase IV trial DUPIREAL [58••], applied these criteria to the real-life data in a large series of patients treated with dupilumab, observing that the criteria were too restrictive at 12 months. The authors demonstrated that the EUFOREA criteria 2021 might lead to the risk of discontinuing the treatment after 1 year in many more patients than those experiencing significant symptoms. Indeed, the established criteria might lead clinicians to wrongly discontinue the treatment even if patient satisfaction is acceptable based on the VAS scores for the main symptoms.

## Discussion

The term “remission” is not new in medicine, especially in rheumatology and oncology [41]. However, at least regarding type 2 conditions, the availability of targeted drugs has raised renewed interest in the remission concept and is paving the way to a new, more comprehensive perspective to look at treatment goals in terms of achievable outcomes and their assessment. It probably reflects the ability of new biologic drugs to specifically interact with pathobiological mechanisms preceding the clinical manifestations, in some cases with the very early phases of the immune cascade, and thus their potential to modify the natural course of the disease even once discontinued [59]. The disease-modifying effect of monoclonal antibodies in type 2 conditions is currently far from being demonstrated [41]. In fact, most of the criteria proposed so far are intended to define on-treatment remission. This is also coherent with the current positioning of biologics, at least in severe asthma and CRSwNP, as an add-on treatment if the traditional treatment is insufficient to achieve disease control.

According to the published evidence focusing on remission in type 2 inflammatory diseases, an unequivocal definition is still lacking. It partially reflects the differences in the currently standardized approach to diagnosis and follow-up, which relies on endoscopy in the case of EoE or lung functional assessment in the case of asthma. However, a full consensus on criteria or cutoffs related to the same disease has not yet been reached, even when comparing definitions. If symptom improvement and clinical evidence of reduced disease activity are part of all the available definitions (Table 4), inflammatory biomarkers do not meet the same agreement.

**Table 4:** Summary of specific tools currently considered in the definition/assessment of remission in type 2 diseases and proposed parameters for remission in chronic rhinosinusitis with nasal polyps (CRSwNP)

	**Atopic dermatitis**	**Eosinophilic esophagitis**	**Asthma**	**CRSwNP**
**Symptoms** (**including patient reported outcomes)**	In children: the absence of signs/symptoms of AD as assessed by a physician (clinical assessment) [11]	NRS dysphagia and odynophagia score ≤ 2 (on a 0–10 scale) on each of the 7 days before the end of the DB [16] EEsAI score ≤ 20 [18]	None in the last 6 months [33], 12 months [30, 34–37], 3 years [29, 31], unspecified period of time [40] ACT < 25 [33] ACQ-6 score < 1.0 or ≤ 0.75 [39] ACT score ≥ 20 [38]	Absence of nasal symptoms (nasal obstruction, smell loss, rhinorrhea, craniofacial algia, etc.). Optional: evaluation of smell function through semi-objective tests
**Health-related questionnaire**	In children: International Study of Asthma and Allergies in Childhood (ISAAC) questionnaire in at least two subsequent follow-up visits covering a period of ≥ 12 months	EEsAI score ≤ 20 [18]	ACT < 25 [33] ACQ-6 score < 1.0 or ≤ 0.75 [39] ACT score ≥ 20 [38]	Evaluation of burden of symptoms on quality of live by SNOT-22 Evaluation of nasal obstruction by VAS or by NCS
**Exacerbation/hospitalization**	AD flares		None in the last 12 months [30, 31, 34–36], 3 years [29]	No exacerbation in the last 12 months (including the absence of evidence of endoscopic recurrence of nasal polyps)
**Functional** (**objective measurements)**			Normal bronchial provocation test [32]; normal lung function at spirometry [33]; prebronchodilator FEV_1_ > 80% predicted [36, 38]; no AHR [37, 40]; no bronchodilatory reversibility; spirometry with FEV_1_ ≥ 80% predicted and FEV_1_/FVC ratio ≥ 0.70 (if not the only factor) [37] Prebronchodilator FEV_1_ increase from baseline of ≥ 100 mL at 6 or 12 months [39]	In CRSwNP, specific functional tests that are routinely adopted are missing. Nevertheless, PNIF and S’S may be useful tools Endoscopic check of the status of the mucosa (NPS and Lund-Kennedy Endoscopy Scale score)
**Biomarkers**		Histologic remission: PEC < 16/mm^2^ [15, 17•] PEC < 5/HPF if combined with low symptomatic scores [16] Eosinophilic Esophagitis Histology Scoring System (EoEHSS)	FeNO < 20 ppb [33] FeNO < 50 ppb [37] Blood eosinophil count of < 300 cells/μL Sputum eosinophil count of < 3% Reduction in subepithelial fibrosis as potential proof of normalized airway pathology [43]	No reliable biomarkers
**Use of topical medication**			None in the last 6 months [33], 12 months [34, 37]), 2 years [32]	Evaluation of intranasal steroid use (including rescue and chronic treatment) in the last 12 months
**Use of oral corticosteroids or systemic corticosteroids**			No oral prednisolone in the last 12 months [38] 2 years [33], unspecified amount of time [40], 6–12 months [39]	Evaluation of systemic steroids intake (including rescue and chronic treatment) in the last 12 months

*ACQ-6* 6-Item Asthma Control Questionnaire, *ACT* Asthma Control Test, *AHR* airway hyperresponsiveness, *EEsAI* Eosinophilic Esophagitis Activity Index, *EoEHSS* Esophagitis Histology Scoring System, *FeNO* fraction of expired nitric oxide, *FEV*_*1*_ forced expiratory flow in 1 s, *FVC* forced vital capacity, *HPF* high-powered field, *NBS* Nasal Blockage Score from 0 (absent) to 3 (severe), *NCS* Nasal Congestion Severity, *NPS* Nasal Polyp Score, *NRS* Numerical Rating Scale, *PEC* peak eosinophil count, *PNIF* peak nasal inspiratory flow, *S’S* Sniffin’ Sticks, *VAS* visual analog scale

In EoE, there are various definitions of histological remission and clinical remission. Although there is general agreement that the definition of histological remission should include low levels of eosinophils per HPF, the threshold can differ, and there is a need for clear criteria regarding the number and consistency of biopsy findings between samples taken from different sites in the esophagus as well as regarding the value of the other histological features typically associated with EoE. Moreover, the definition of clinical remission as well as the instruments regarding its assessment is still matter of debate, with further research needed to better define it.

In asthma, most definitions of remission require the absence of asthma symptoms (i.e., wheezing, chest tightness, and shortness of breath), flares or exacerbations, in addition to the absence of AHR or spirometry evidence of obstruction. Inflammatory biomarkers are included as an accepted component of some definitions of remission only. Although studies vary on whether or not this definition applies during asthma treatment, there does appear to be a consensus that patients must be free of systemic corticosteroid use to be considered in remission. The consensus report by Menzies-Gow and colleagues provides a framework for the definition of remission in asthma [40], but this definition has been criticized for being “lenient” on short-acting β-agonist (SABA) use since SABA use can be a surrogate marker for symptoms [43].

However, a multidimensional evaluation should be assessed when considering complete remission, including clinical, inflammatory, and histologic criteria. How to combine or tailor the three perspectives according to disease severity at baseline or timing of assessment of treatment category has not yet reached a consensus. On a practical ground, the poor agreement on inflammatory biomarkers might be related to the difficulties in their assessment in non-specialized centers and the complexity of their correct interpretation.

In the case of CRSwNP, no criteria clearly related to the remission concept have been proposed so far. However, in light of the availability of new biologic drugs significantly impacting CRSwNP symptoms and endoscopic findings, a clear definition of remission would help clinicians set therapeutic algorithms, especially for the long-term management of patients.

Combining the evidence from the clinical trials on biologics in CRSwNP and the available definitions of remission in other type 2 inflammatory diseases (Table 5), we now present some proposals for defining on-treatment remission in CRSwNP. In Table 5, we also listed specific tools that may be useful to refine the definition of remission and its assessment, and maybe strive towards creating validated cutoff values in the future.

**Table 5:** Definition of disease control in chronic rhinosinusitis according to the European Position Paper on Rhinosinusitis and Nasal Polyps [60]

**Item**	**Definition for control** (**all must be present)**
Nasal blockage	Not present or bothersome
Rhinorrhea/postnasal drip	Little or mucous
Facial pain/pressure	Not present or bothersome
Smell	Normal or only slightly impaired
Sleep disturbance or fatigue	Not present or bothersome
Nasal endoscopy	Healthy or almost healthy
Rescue treatment in the last 6 months	Not needed

We suggest defining remission starting from the approach taken in asthma and EoE, in which the definition of remission includes the resolution of symptoms and improvements in objective parameters of disease severity and/or inflammatory activity. We also believe that any definition of remission in patients with CRSwNP should be consistent with existing regulatory requirements, as well as being feasible for use in routine clinical practice.

Regarding objective parameters of disease severity and/or local inflammatory activity, endoscopy is the preferred method of assessing disease activity and is consistent with the US FDA and EUFOREA guidance to assess change in NPS [56, 57•] and the EPOS criteria to show healthy nasal tissue as a measure of disease control in CRS (Table 4) [60]. Measures based on CT imaging studies are less practical in routine clinical practice, particularly in resource-limited settings [61].

**Fig. 1:**
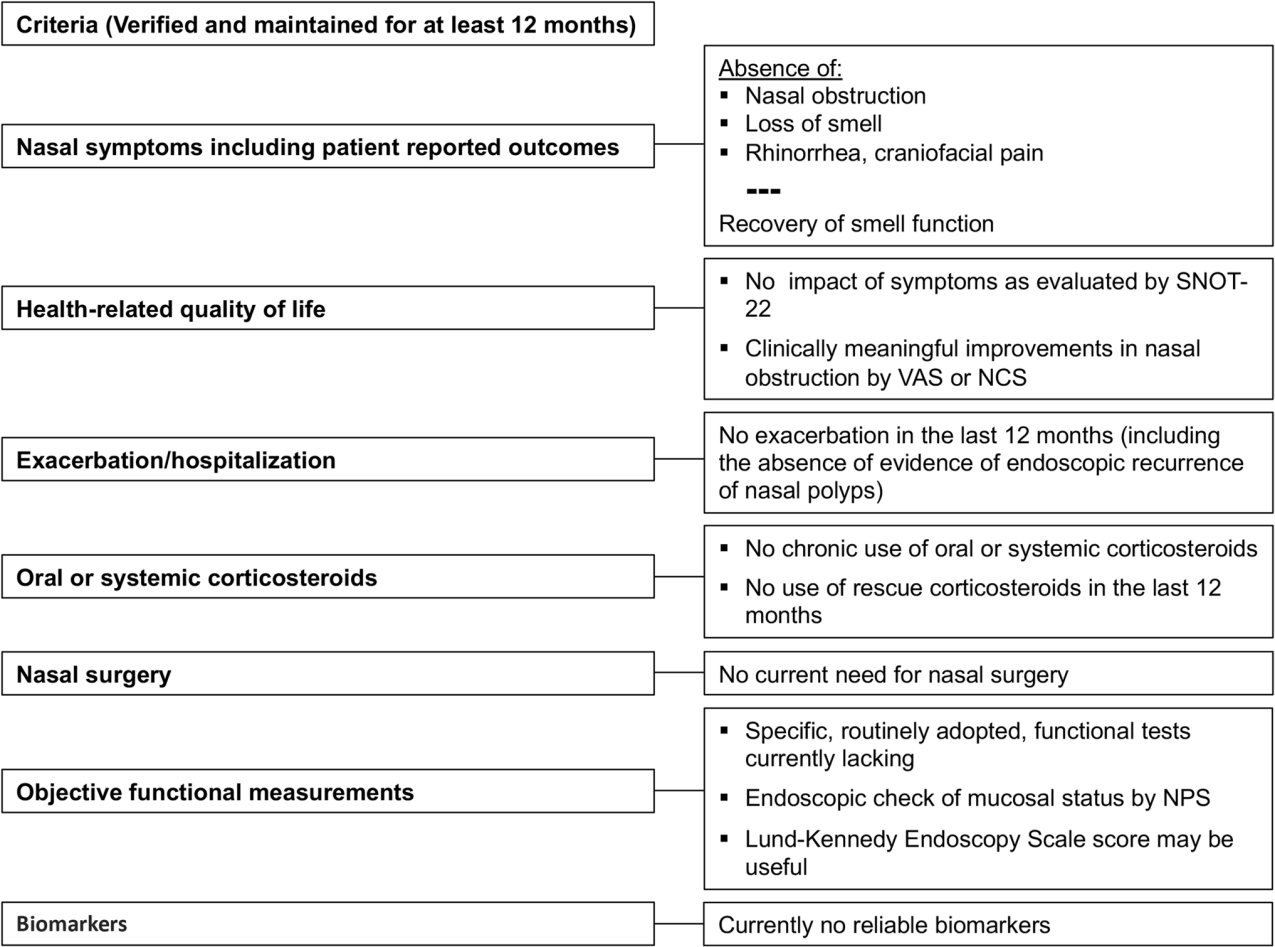
Proposed criteria defining remission in chronic rhinosinusitis with nasal polyps (CRSwNP). *NCS* Nasal Congestion Severity, *NPS* Nasal Polyp Score, *SNOT-22* 22-Item Sino-Nasal Outcome Test, *VAS* visual analog scale

Finally, we believe that the definition of remission in CRSwNP should include the following criteria, all of them to be verified and maintained for a period of at least 12 months: absence of symptoms (nasal obstruction, loss of smell, rhinorrhea as the main ones); no impact of symptoms on quality of life; no need of surgery; no chronic or rescue systemic steroids or antibiotics; and recovery of smell function, possibly evaluated by objective test. Our proposal for defining remission is summarized in Fig. 1.

Normal nasal respiratory function, and endoscopic evidence of healthy mucosa nasal cytology, is not included in our proposal, although some evidence sustains its validity within the diagnostic workup and differential diagnosis of rhinitis. In fact, we referred to the on-treatment remission concept, the term treatment including both traditional nasal steroid therapy and biologic drugs. Even in the case of the last, as the current positioning of targeted therapy is as an “add-on” to traditional drugs, patients are expected to take topical steroids, which definitely impair the accuracy of nasal cytology independently of whether remission is achieved or not [62].

Besides our proposal, an agreement should be reached in the future to define specific outcomes and cutoffs that should be considered in the definition of remission in CRSwNP.

A further point of discussion is related to the high frequency of coexisting severe asthma and CRSwNP, which expresses a demonstrated common pathobiological background [63]. It is also well known that patients suffering from both conditions experience a higher disease burden. In that light, a definition of remission combining an integrated evaluation of upper and lower airways should probably be applied.

In addition, under a broader view, the recent advances in the pathobiology of type 2 inflammatory conditions suggest the so-called epithelial barrier dysfunction as a common immunological background [64, 65]. Although not easy to be clearly identified or assessed in clinical practice, it sustains the idea that a definition of remission restricted to one single organ or condition might not sufficiently accurately reflect the disease’s systemic background. A more global multidimensional definition of remission should probably be considered, according to the patient clinical profile.

## Conclusions

Based on the evolving understanding of remission in asthma (and to a lesser extent in other type 2 inflammatory diseases), we propose a definition of remission in patients with CRSwNP that incorporates symptomatic improvement as well as objective evidence of improvement in the underlying disease severity (Fig. 1). Future studies and consensuses should propose validated criteria with cutoffs for the day-to-day definition of remission for CRSwNP.

Under a broader view, a definition of remission not restricted to one single organ or condition should be evaluated on patients suffering from coexisting type 2 inflammatory diseases.

## Data Availability

The data that support the findings of this study are available for this journal.
